# Statistical Assessment of Depth Normalization for Small RNA Sequencing

**DOI:** 10.1200/CCI.19.00118

**Published:** 2020-06-29

**Authors:** Li-Xuan Qin, Jian Zou, Jiejun Shi, Ann Lee, Aleksandra Mihailovic, Thalia A. Farazi, Thomas Tuschl, Samuel Singer

**Affiliations:** ^1^ Department of Epidemiology and Biostatistics, Memorial Sloan Kettering Cancer Center, New York, NY; ^2^ Department of Surgery, Memorial Sloan Kettering Cancer Center, New York, NY; ^3^ Laboratory of RNA Molecular Biology, The Rockefeller University, New York, NY

## Abstract

**PURPOSE:**

Methods for depth normalization have been assessed primarily with simulated data or cell-line–mixture data. There is a pressing need for benchmark data enabling a more realistic and objective assessment, especially in the context of small RNA sequencing.

**METHODS:**

We collected a unique pair of microRNA sequencing data sets for the same set of tumor samples; one data set was collected with and the other without uniform handling and balanced design. The former provided a benchmark for evaluating evidence of differential expression and the latter served as a test bed for normalization. Next, we developed a data perturbation algorithm to simulate additional data set pairs. Last, we assembled a set of computational tools to visualize and quantify the assessment.

**RESULTS:**

We validated the quality of the benchmark data and showed the need for normalization of the test data. For illustration, we applied the data and tools to assess the performance of 9 existing normalization methods. Among them, trimmed mean of M-values was a better scaling method, whereas the median and the upper quartiles were consistently the worst performers; one variation of remove unwanted variation had the best chance of capturing true positives but at the cost of increased false positives. In general, these methods were, at best, moderately helpful when the level of differential expression was extensive and asymmetric.

**CONCLUSION:**

Our study (1) provides the much-needed benchmark data and computational tools for assessing depth normalization, (2) shows the dependence of normalization performance on the underlying pattern of differential expression, and (3) calls for continued research efforts to develop more effective normalization methods.

## INTRODUCTION

Several analytic methods have been proposed for normalizing sequencing depth. Earlier methods were based mostly on the scaling strategy, which calculates a scaling factor (eg, the total number of counts) for each sample to adjust the data.^1-3^ Later, more-involved methods based on regression (eg, with regard to selected principal components of all or some markers) were proposed on the basis of empirical observations that depth does not influence sequencing data in a simple overall shifting manner and concerns that it can be complicated by other nonspecific sources of handling variations.^4-6^ Many of these methods were developed in the context of differential expression analysis, and their performance has been assessed mostly using parametrically simulated data and/or cell-line–mixture data that may not realistically reflect the distributional characteristics of sequencing data.^1,2,4^

**CONTEXT**

**Key Objective**
There is a pressing need for objective assessment of depth normalization methods using realistic and robust benchmark data in the context of small RNA sequencing.
**Knowledge Generated**
We collected such benchmark data and accompanying test data and developed computational tools. We demonstrated their use with the assessment of 9 normalization methods. These methods were, at best, moderately helpful, especially when differential expression was extensive and asymmetric.
**Relevance**
Our study provides evidence on the subpar performance of existing normalization methods and calls for continued research efforts to develop more effective methods.

We set out to develop the data and analytics to enable a more realistic and objective assessment of depth normalization methods, focusing on a class of small RNAs called microRNAs (miRNAs). MiRNAs are 18 to 22 nucleotides long, which minimizes the potential bias in abundance estimation due to gene length variation, as seen in RNAs.^7,8^ They play an important regulatory role in gene expression in the cell and are closely linked to cell apoptosis and carcinogenesis.^9,10^

Toward this end, we designed and collected a unique pair of data sets for the same set of tumor samples at Memorial Sloan Kettering Cancer Center (MSKCC). The first data set was collected using (1) uniform handling to minimize data artifacts and (2) balanced sample-to-library assignment (via blocking and randomization) to avoid confounding for any residual artifacts with the sample groups under comparison. For the same set of samples, a second data set was collected without using such careful study design, resulting in obscuring depth variations. Evidence of differential expression was assessed in the first data set, serving as the benchmark; normalization methods were tested in the second, followed by differential expression assessment and benchmark comparison. Throughout the rest of this article, we refer to the first data set as the benchmark data set and the second as the test data set. For extra quality assurance, we added for the benchmark data set two pooled samples shared across all libraries and 10 prevalidated calibrators added at fixed concentrations.

In addition to the empirical data, we devised a data perturbation strategy to generate additional data set pairs under various differential expression scenarios. Furthermore, we put together a set of graphical tools and numeric metrics for visualizing and quantifying the impact of depth normalization on differential expression detection. Here, we report the data and tools and illustrate their use on nine normalization methods that are relatively commonly used in the literature.^1,2^

## METHODS

### Tumor Sample Preparation and microRNA Sequencing

Myxofibrosarcoma (MXF) and pleomorphic malignant fibrous histiocytoma (PMFH) are the two most common and aggressive subtypes of genetically complex soft tissue sarcoma.^11-13^ Their tumor samples are typically large enough for RNA extraction with sufficient quantity and quality for sequencing. In this study, we used 27 MXF samples and 27 PMFH samples, which were all from newly diagnosed, previously untreated, primary tumors collected at MSKCC between 2000 and 2012. Sample preparation and extraction were performed in the Singer laboratory at MSKCC. Library preparation and sequencing were done in the Tuschl laboratory at Rockefeller University. Detailed description of these steps was previously reported.^14,15^

### Design and Analysis of the Benchmark Data

#### Study design.

With barcode multiplexing, we used three libraries to sequence the 54 individual tumor samples and two pooled samples (one from pooling the 27 MXF samples and the other from pooling the 27 PMFH samples); each library included nine MXF samples, nine PMFH samples, the pooled MXF sample, and the pooled PMFH sample. We carefully planned our study for generating this benchmark data set so the library preparation and read capture were each processed by a single experienced technician in one run. In addition, samples of each tumor type were randomly assigned to the three libraries and the 20 barcodes. To further ensure data quality, we included 10 calibrators with fixed input concentrations for each sample to use as negative controls for differential expression.^14^

#### Quality assessment analysis.

We assessed the agreement of the three sequencing runs for each pooled sample, as well as the agreement between group means based on individual samples and those based on pooled samples, using scatter plots and concordance correlation coefficients.^16^

#### Differential expression analysis.

We assessed the evidence against the null hypothesis of equivalent expression in MXF versus PMFH using the benchmark data for the 54 tumor samples. We used voom (as implemented in the limma package in R; R Foundation, https://www.r-project.org/about.html) as the primary method for differential expression analysis, with the results reported in this article; and *edgeR* as a secondary method with the results reported in the Data Supplement.^17-19^ Additional description of the choice of these methods is provided in the Appendix.

### Design and Analysis of the Test Data

#### Study design.

miRNAs for the same 54 tumors used for the benchmark data were resequenced using neither uniform handling nor balanced library assignment. In this second study, these samples were sequenced in the order of sample collection and processed in multiple runs. Care was taken to ensure consistent sample handling and RNAs used for sequencing runs of the same sample were derived from the same cryomold.

#### Depth normalization.

We examined nine normalization methods, including six scaling-based methods and three regression-based methods (six, if counting the variations for two of the methods). The former included total count (TC),^2^ upper quartile (UQ),^1^ median,^2^ trimmed mean of M values (TMM),^17^ DESeq,^3^ and PoissonSeq.^20^ The latter included quantile normalization (QN) with and without removing low-count miRNAs,^21^ surrogate variable analysis (SVA),^6^ and remove unwanted variation (RUV), with three variations: RUVg, RUVr, and RUVs.^5^ Detailed description of these methods is provided in the Appendix.

#### Differential expression analysis.

The test data were assessed for differential expression, before and after normalization, using voom as the primary method and edgeR as the secondary method in the same manner as the benchmark data.

### Computational Tools for Evaluating Depth Normalization

We assembled several useful graphical tools and numeric measures to evaluate the impact of a normalization method on the overall distribution of the test data and on the differential expression status and significance ranking of miRNAs, as well as to explore the relationship among different normalization methods.

#### Relative log expression plot.

The relative log expression plot examines the impact of normalization on the overall data distribution.^22^ More specifically, it adjusts the log2 count data for each sample by subtracting its median and displays the deviations as a box plot. In our implementation, we sorted the box plots for the samples within each group by the upper quartiles of their deviations determined in the benchmark data.

#### Concordance at the top plot.

The concordance at the top (CAT) plot compares the ranking of significant miRNAs on the basis of the test-data *P* values with the benchmark.^6^ More specifically, it uses indices 1 to K as the *x*-axis, and the percentage of agreement in the miRNAs ranked among the top K between the two data sets as the *y*-axis. The plot can be done for multiple normalization methods at a time, with each method plotted in a different color.

#### Venn diagram.

The *P* values from differential expression analysis were used to derive a marker set at a given significance level: markers with *P* values smaller than the significance level were declared differentially expressed, and those having larger *P* values were declared nondifferentially expressed. Differential expression statuses were compared between the two data sets graphically via the Venn diagram.

#### False-negative rate and false-discovery rate.

The comparison of differential expression statuses between the two data sets was summarized numerically using a false-negative rate (FNR) and a false-discovery rate (FDR), at the risk of abusing the terminology. They were compared between different normalization methods, using the scatter plot.

#### Dendrogram showing the clustering of normalization methods.

To assess similarity between normalization methods, their test-data *P* values (on the −log10 scale) were subjected to hierarchical clustering using the Euclidean distance and the Ward linkage. Clustering results were displayed using the dendrogram.

### Data Perturbation to Simulate Additional Data Set Pairs

#### Overall strategy.

We examined the normalization methods in additional data sets simulated under a range of differential expression scenarios. More specifically, the sample-type labels were permuted to reach a specific proportion of differential expression and a specific magnitude of median group-mean difference (for log2 count) in the benchmark data set, and the same permutation of group labels was then applied to the test data. The goal was to generate data sets with various proportions of differentially expressed markers (eg, 2%, 10%, 20%) and magnitudes of mean difference among differentially expressed markers. This strategy allowed us to maintain the between-miRNA correlation structure in each data set and not make any parametric assumptions regarding the distributions of sequencing count and depth variation.

#### Perturbation steps.

Data were simulated with a combination of hierarchical clustering and random shuffling, with stratification by library (ie, an equal number of samples in each group was allocated to each library), in the following three steps: (1) the benchmark data of the 54 samples were clustered using K-means clustering to two clusters (via the *pam()* function in the *cluster* library) and labeled as groups 1 and 2; (2) nine seed samples were randomly selected from each group, with three samples from each sequencing library; (3) the remaining 36 samples were then randomly and equally allocated to the two groups. For each permutation of the 54 samples, differential expression between the two groups was assessed with regard to the proportion of differential expression and the magnitude in terms of the median of marker-specific group-mean differences.

#### Simulation scenarios.

Four scenarios of differential expression were examined. The first three scenarios each had 100 data sets randomly selected, and the last scenario had 39 data sets.

1. Limited and symmetric: 2% (1.75%-2.25%, to be more precise) differential expression with group mean difference approximately 0 (ranging from −0.5 to 0.5);
2. Limited and asymmetric: 2% differential expression with group mean difference approximately 3 (2-4);
3. Moderate and asymmetric: 10% (8%-12%) differential expression with group mean difference approximately 3; and
4. Extensive and asymmetric: 20% (15%-25%) differential expression with group mean difference approximately 3.

#### Analysis of simulated data.

Each pair of the simulated data sets was analyzed and compared in the same manner as the empirical data. The results, in terms of FNR and FDR, for each simulation scenario were displayed aggregately as box plots.

## RESULTS

### Quality Assessment of the Benchmark Data

**FIG 1.**
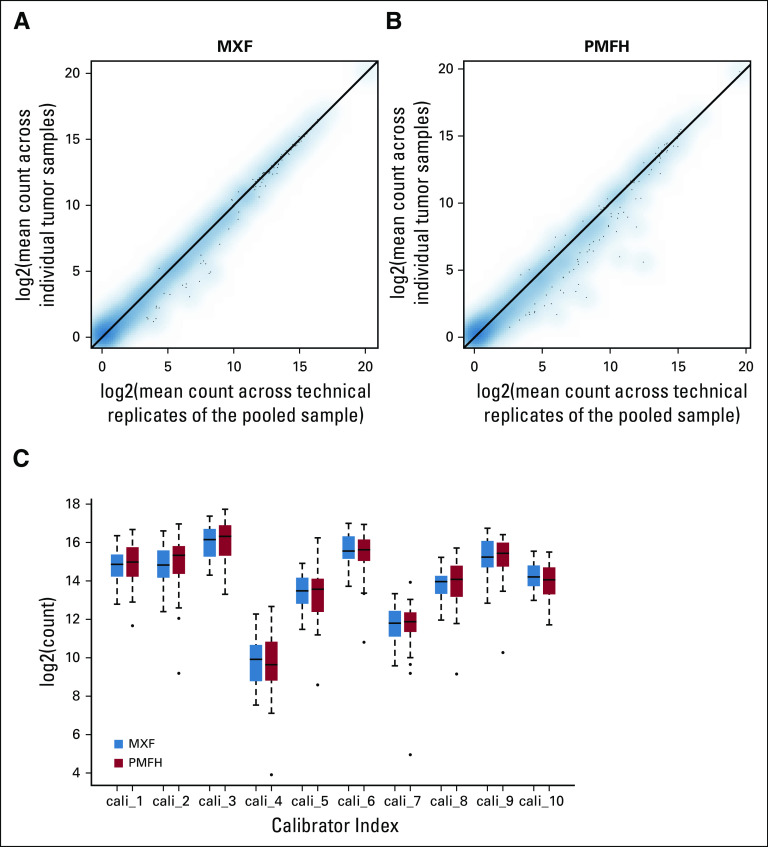
(A) Scatter plot of group means (for log2 counts) estimated with individual myxofibrosarcoma (MXF) tumors versus group means estimated with the three technical replicates for the pooled MXF sample. Each data point represents an miRNA. (B) Scatter plot of group means (for log2 counts) estimated with individual pleomorphic malignant fibrous histiocytoma (PMFH) tumors versus group means estimated with the three technical replicates for the pooled PMFH sample. Each data point represents an miRNA. (C) Box plot for the 10 calibrators by sample type in the benchmark data.

**FIG A1.**
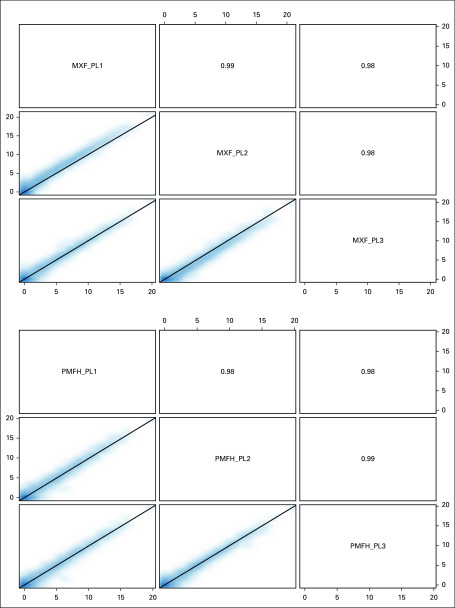
Scatter plot between triplicate runs of the pooled myxofibrosarcoma sample (MXF_PL) and between triplicate runs of the pooled pleomorphic malignant fibrous histiocytoma sample (PMFH_PL). Each data point represents a microRNA.

There was excellent agreement between the replicates for each pooled sample (concordance correlation coefficient: 0.97 to 0.99; Appendix Fig A1). In addition, group means estimated by the 54 tumors highly agreed with those estimated by the triplicates (concordance correlation coefficient: 0.99), indicating the consistency of the averaged sequencing data and the data of the pooled samples (Fig 1A and 1B). Furthermore, all 10 calibrators showed equivalent expression between the 2 sample groups (mean difference, −0.03 to 0.09; *P*-value range, 0.11 to 0.96; Fig 1C). These observations collectively confirmed the quality of the benchmark data.

### Differential Expression Analysis of the Benchmark Data

**FIG 2.**
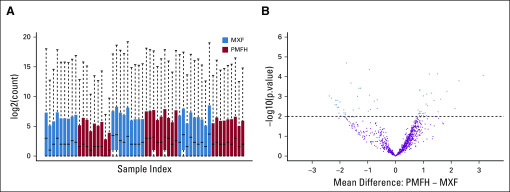
(A) Box plot of the benchmark data with one box per sample. The boxes were sorted by sequencing libraries and colored by sample type. (B) Volcano plot for the differential expression analysis using voom for the benchmark data. MXF, myxofibrosarcoma; PMFH, pleomorphic malignant fibrous histiocytoma.

Figure 2A shows an overview of the count distribution for the benchmark data on the log2 scale. Among the 1,033 miRNAs in the data, 59 (6%) were differentially expressed at a *P*-value cut off of .01 (Fig 2B). By chance alone, there could only be 10 miRNAs with *P* < .01. The average count of these 59 miRNAs ranged from a few to several hundreds of thousands, with the mean differences between MXF and PMFH ranging from 1.4- to 8-fold (Appendix FigA2A-A2C).

### Differential Expression Analysis of the Test Data

**FIG 3.**
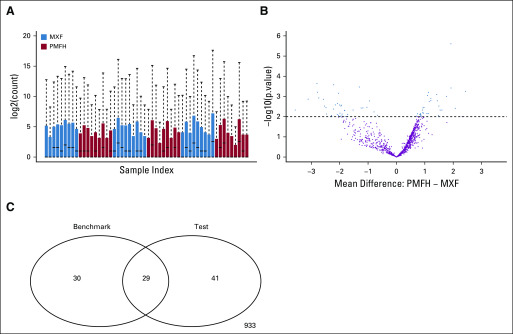
(A) Box plot of the test data with 1 box per sample. The boxes were colored by sample type and sorted on the basis of their order in the benchmark data. (B) Volcano plot for the differential expression analysis using voom for the test data without any depth normalization. (C) Venn diagram comparing the number of significant microRNAs identified by the benchmark data and by the test data. 933 is the number of markers identified as nonsignificant in both the test and benchmark sets.

**FIG A3.**
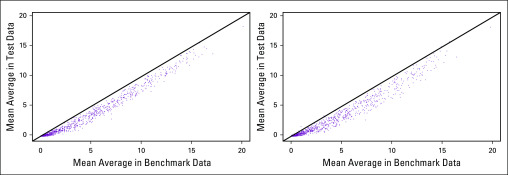
(A) Scatter plot of group means for myxofibrosarcoma (MXF) in the test data versus that in the benchmark data. Each data point represents a microRNA. (B) Scatter plot of group means for pleomorphic malignant fibrous histiocytoma (PMFH) in the test data versus that in the benchmark data. Each data point represents a microRNA.

As shown in Fig 3A, the test data possessed more systematic variations than the benchmark data. Without normalization, 70 miRNAs (7%) were identified as differentially expressed (Fig 3B). Twenty-nine of these 70 miRNAs were claimed to be differentially expressed by the benchmark data, resulting in an FNR of 30 of 59 (51%) and an FDR of 41 of 70 (59%; Fig 3C). Appendix Figure A3A and A3B compares the estimated group means in the test data with those in the benchmark data.

### Evaluation of Depth Normalization Using the Empirical Data

**FIG 4.**
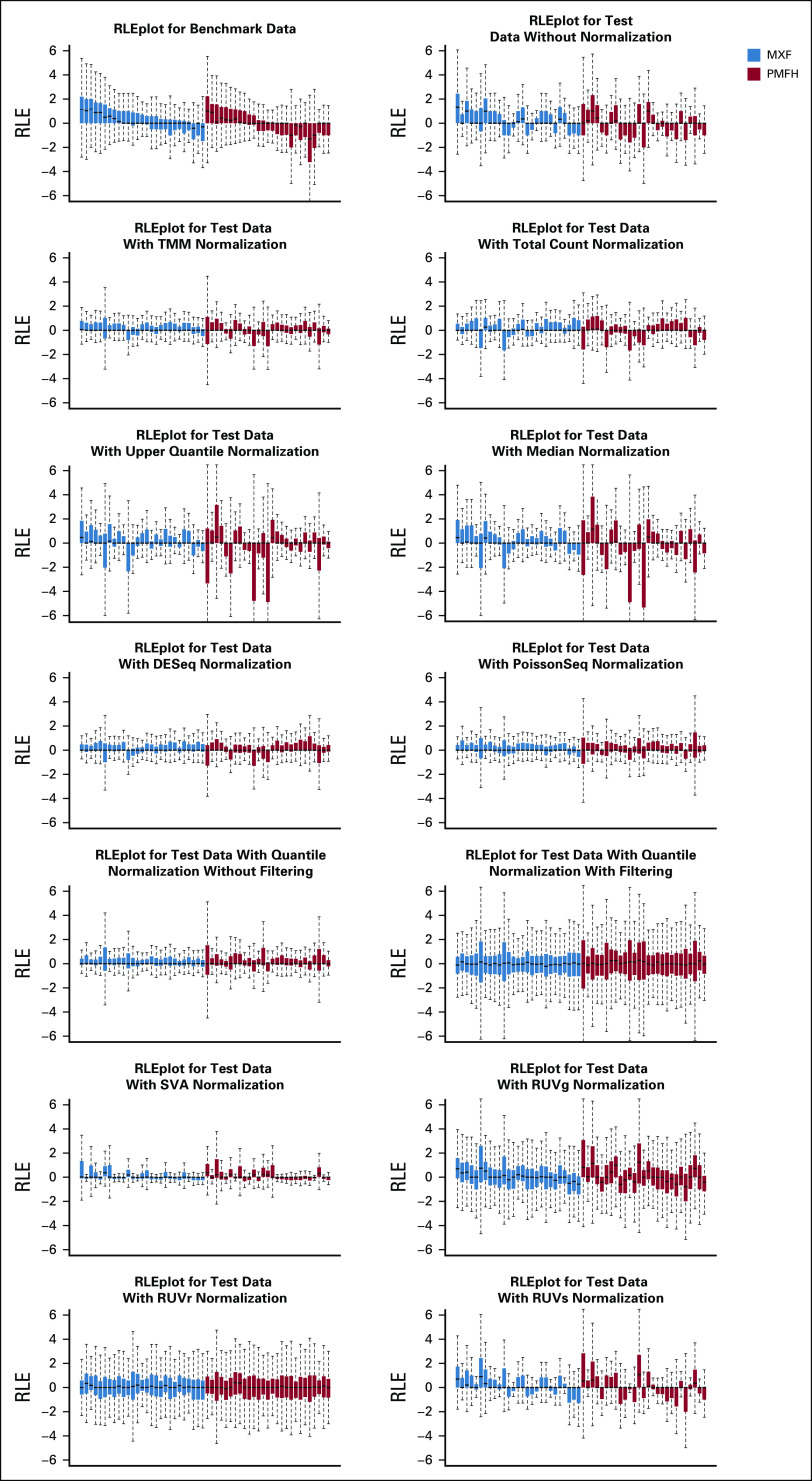
Relative log expression (RLE) plots for benchmark data, test data, and test data after normalization with various methods. Samples were ordered by their residuals’ upper quartiles in the benchmark data. RUV, remove unwanted variation; SVA, surrogate variable analysis.

**FIG 5.**
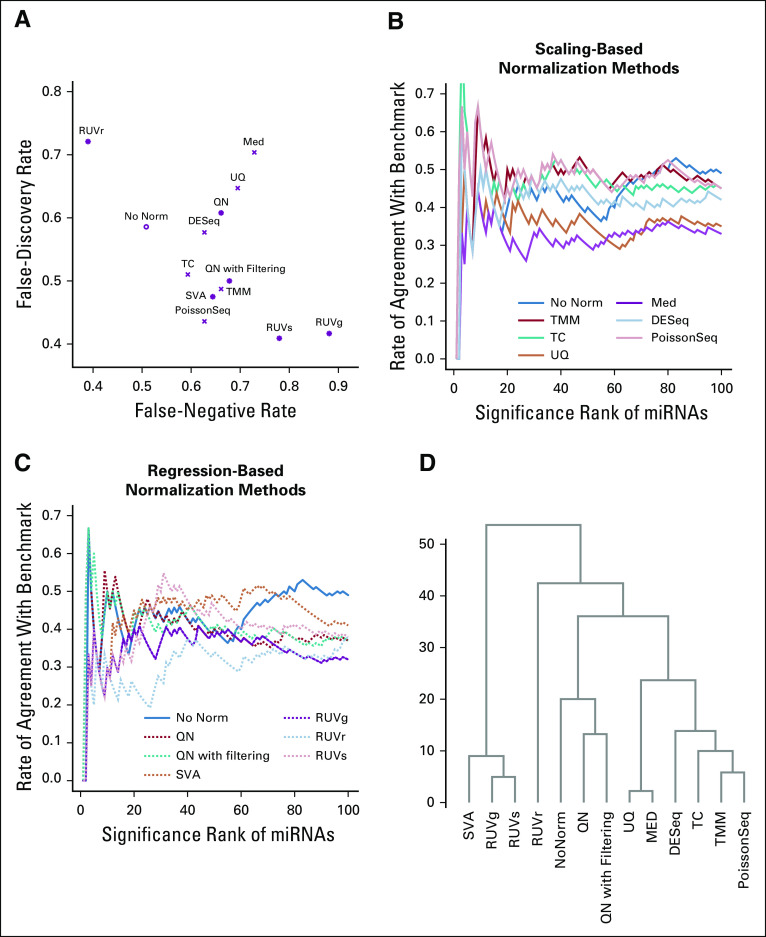
(A) Scatter plot comparing false-negative rate versus false-discovery rate for the test data before and after normalization. (B) CAT plot comparing the agreement of significance ranking in the test data (before and after scaling normalization) with that in the benchmark data. (C) CAT plot comparing the agreement of significance ranking in the test data (before and after regression-based normalization) with that in the benchmark data. (D) Dendrogram comparing the *P* values (on the −log10 scale) for test data after normalization by different methods. CAT, concordance at the top; Med, median; miRNA, microRNA; No Norm, no normalization; QN, quantile normalization; RUV, remove unwanted variation; TC, total count; TMM, trimmed mean of M-values; UQ, upper quartile.

**FIG A4.**
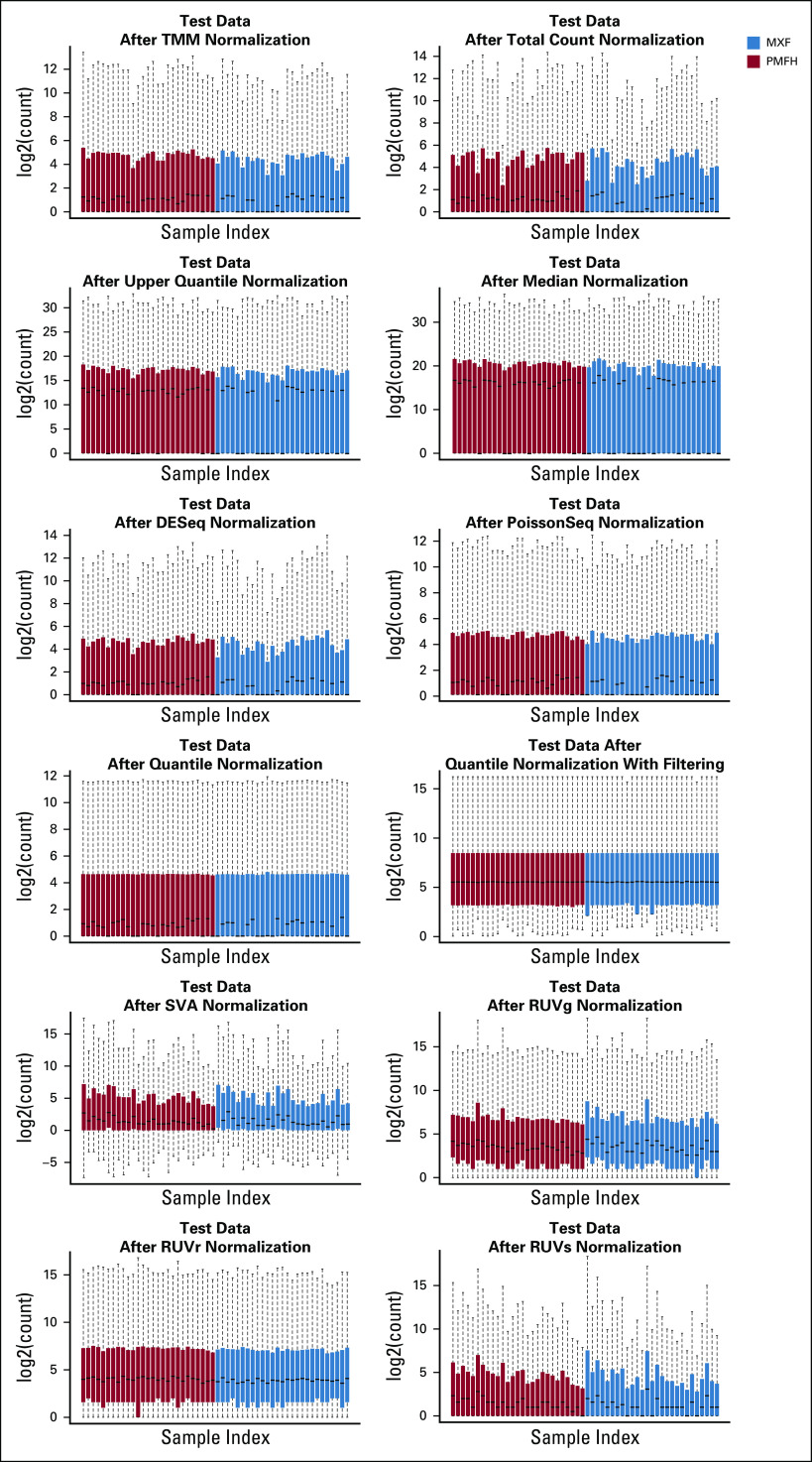
Box plots of test data after normalization. MXF, myxofibrosarcoma; PMFH, pleomorphic malignant fibrous histiocytoma.

The effects of normalization on the overall distribution of the test data are shown in Figure 4 and Appendix Figure A4. Figure 5A presents the impact of normalization on significance detection in terms of FNR and FDR. With scaling-based normalization, the number of differentially expressed miRNAs decreased to 49 for TC (FNR: 59%; FDR: 51%); 51 for UQ (FNR: 69%; FDR: 65%); 54 for median (FNR: 73%; FDR: 70%); 51 for TMM (FNR: 69%; FDR: 65%); 52 for DEseq (FNR: 63%; FDR: 58%); and 39 for PoissonSeq (FNR: 63%; FDR: 44%). With regression-based normalization, the number of differentially expressed miRNAs decreased to 40 for SVA (FNR: 64%; FDR: 48%); 51 for QN (FNR: 66%; FDR: 61%); 38 for QN with filtering (FNR: 68%; FDR: 50%); 22 for RUVs (FNR: 78%; FDR: 41%); and 12 for RUVg (FNR: 88%; FDR: 42%). The number of differentially expressed miRNAs increased to 129 (FNR: 39%; FDR: 72%) when using RUVr. Hence, in the analysis of our data, normalization methods such as TC, PoissonSeq, SVA, RUVs, and RUVg decreased the FDR at the price of increased numbers of missed positives; RUVr decreased the number of missed positives at the price of increased FDRs; other methods increased both. All in all, normalization did not seem to effectively improve the results of significance detection in these data.

We then evaluated how faithfully miRNAs were ranked on the basis of the significance level using CAT plots. Among scaling-based normalization methods, TMM and PoissonSeq outperformed no normalization up to the top 70 ranked miRNAs, with approximately 50% of the top-ranked miRNAs the same as the benchmark; TC outperformed no normalization for the top two miRNAs and then from approximately the top 20 to the top 70 miRNAs; DESeq was comparable to no normalization throughout the range we examined (ie, up to the top 100 miRNAs); median and UQ were consistently the worst performers (Fig 5B). Among regression-based normalization methods, SVA outperformed no normalization from approximately the top 15 to the top 70 ranked miRNAs, with approximately 40% to 50% agreement to the benchmark; QN was comparable to no normalization among the top 50 ranked miRNAs; RUVg and RUVr consistently performed worse than no normalization (Fig 5C).

We additionally compared normalization methods by clustering their *P* values. The comparison showed separation of scaling methods from regression-based methods and separation of median and UQ from the other scaling methods (Fig 5D).

**FIG A5.**
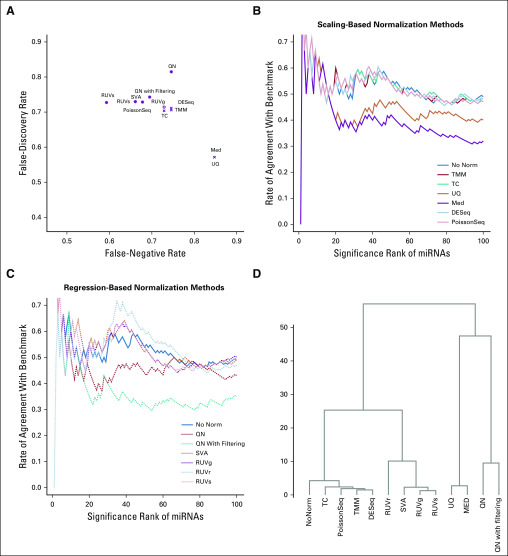
(A) Scatter plot comparing the false-negative rate with the false-discovery rate for test data before and after normalization, using *edgeR* as the method for differential expression. Each data point represents a normalization method. (B) CAT plot comparing the agreement of significance ranking of top microRNAs (miRNAs) in the test data (before and after scaling normalization) with that in the benchmark data, using *edgeR* as the method for differential expression. (C) CAT plot comparing the agreement of significance ranking of top miRNAs in the test data (before and after regression-based normalization) with that in the benchmark data, using *edgeR* as the method for differential expression. (D) Dendrogram comparing the *P* values (on the −log10 scale) for test data among different methods for normalization using *edgeR* as the method for differential expression. CAT, concordance at the top; Med, median; No Norm, no normalization; QN, quantile normalization; RUV, remove unwanted variation; TC, total count; TMM, trimmed mean of M-values; UQ, upper quartile.

We repeated our evaluation using edgeR as the method for differential expression analysis and observed similar results (Appendix Fig A5A-A5D).

### Evaluation of Depth Normalization Using Simulated Data

**FIG 6.**
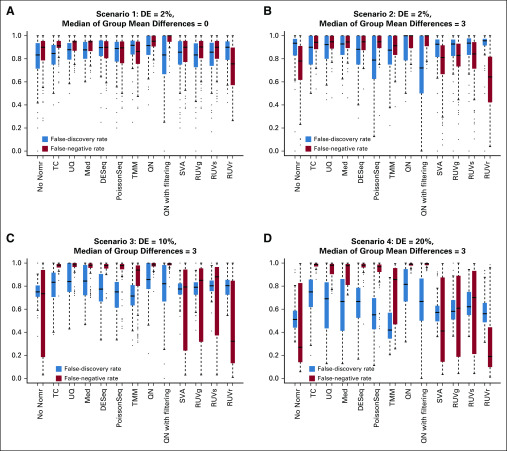
Box plot of false-negative rate and false-discovery rate from the simulation study under four scenarios of differential expression (DE): (A) low and symmetric; (B) low and asymmetric; (C) moderate (10%) and asymmetric; and (D) moderate (20%) and asymmetric. Med, median; No Norm, no normalization; QN, quantile normalization; RUV, remove unwanted variation; TC, total count; TMM, trimmed mean of M-values; UQ, upper quartile.

Results from the simulation study are shown in Figure 6. When differential expression in the data was low (2%) and symmetric, most methods did not seem to help decrease the FNR or FDR; QN with filtering tended to decrease the FDR and, at the same time, increase the FNR, whereas RUVr tended to decrease the FNR and increase the FDR, similar to how they behaved in the empirical data.

When differential expression was low (2%) and asymmetric, TMM, TC, and PoissonSeq occasionally decreased the FDR, yet frequently increased FNR; again, QN with filtering tended to decrease the FDR and increase the FNR, whereas RUVr tended to decrease the FNR and increase the FDR; the other methods decreased neither the FNR nor the FDR.

When differential expression in the data was moderate (ie, 10% and 20%) and asymmetric, test data with no normalization were often associated with a reasonable FDR and FNR, due to the strong level of biologic signal; TMM occasionally decreased the FDR yet frequently increased the FNR, whereas the other scaling normalization methods consistently increased both the FDR and the FNR; RUVr tended to decrease the FNR but not the FDR.

Taken together, the performance of normalization methods depended on the specific pattern of differential expression and, in general, only brought limited benefits; TMM tended to outperform the other scaling methods, and RUVr tended to outperform the other regression-based methods; the median and UQ methods were consistently the worst performers among the methods examined in our study.

## DISCUSSION

In absence of a predetermined standard for comparison, authors of a new normalization method have the freedom to select evaluation procedures that favor their method and subsequently claim superiority over other methods. This self-assessment trap results in contradictory information for users deciding on a method. In this study, we addressed this problem for miRNA sequencing by developing the carefully designed pair of data sets, the resampling-based simulation algorithm, and the relevant graphical and numeric analytics. We make these data and tools publicly available to the research community so interested researchers can reproduce our study and study additional methods reported in the literature and new methods as they emerge.

We previously successfully applied the paired data set strategy to assess normalization methods for miRNA microarrays.^11,23-25^ We expect this general strategy to be useful in assessing normalization for other types of high-throughput data, as well. In this article, we used this strategy to examine the unique challenge of depth normalization for miRNA sequencing. Our findings can provide a useful stepping stone for understanding the same issue of depth normalization for the sequencing of RNAs and metagenomics.^26,27^ Compared with miRNA sequencing, RNA sequencing is subject to an additional source of depth variation from gene length, whereas metagenomic sequencing faces the complication due to data compositionality and sparsity to a greater extent.^28^ They present even more challenging scenarios to have effective normalization methods.

In conclusion, caution should be exercised when applying depth normalization, and more effective methods should be developed with robust, realistic, and objective assessment.

## Figures and Tables

**FIG A2.**
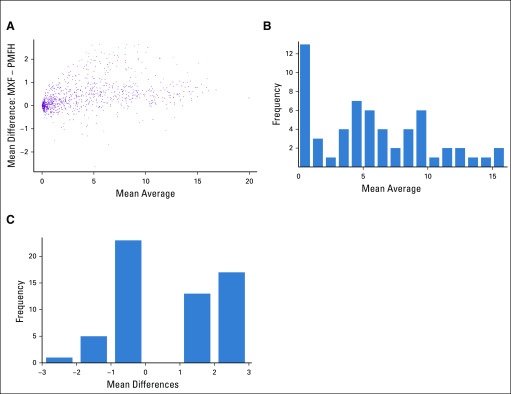
(A) Scatter plot of group mean differences versus group mean averages in the benchmark data, with the significantly differentially expressed microRNAs (miRNAs) highlighted in red. Each data point represents an miRNA. (B) Histogram of group mean averages across 54 samples for significant miRNAs in the benchmark data. (C) Histogram of group mean differences between MXF and PMFH for significant miRNAs in the benchmark data.

## Appendix

### A. Method 1: Choice of Methods for Differential Expression Analysis

We assessed the evidence against the null hypothesis of equivalent expression in myxofibrosarcoma versus pleomorphic malignant fibrous histiocytoma using the benchmark data for the 54 tumor samples. Currently there are 3 state-of-the-art statistical tests for differential expression analysis of sequencing data: (1) edgeR, (2) DESeq, and (3) voom. Among them, edgeR and DESeq assume a negative binomial distribution for the data across samples, whereas voom assumes a normal distribution for the logarithm-transformed data. We previously conducted an empirical comparison of the three methods using two publicly available data sets, one from The Cancer Genome Atlas ovarian study and the other from a breast cancer study. In this previous study, we found the *P* values based on the three methods were close, with voom providing a middle ground between edgeR and DESeq; in addition, among the microRNAs (miRNAs) whose differential expression status differed between the methods, voom was more aligned with the empirical evidence. As such, in this article, we used voom as the primary method for differential expression analysis with the results reported in the main text, and edgeR as a secondary method with the results reported in the Data Supplement.

### B. Method 2: Descriptions of Depth Normalization Methods Evaluated

We examined nine commonly used normalization methods, including six scaling-based methods and three regression-based methods (6 if counting the variations for two of the methods). A scaling-based method calculates a scaling factor on the basis of the data for each sample and divides its counts by this factor. A regression-based method can be nonparametric or parametric: nonparametric methods are based on, for example, a quantile-quantile plot, whereas parametric methods are based on a linear regression, which typically includes a covariate representing systematic depth variation (by using a known batch variable or deriving a surrogate batch variable from the data) in a regression framework for analysis of differential expression.

1.Total count (TC) calculates the total count of mappable reads in each sample and uses its ratio over the average total count across samples as the scaling factor. It has also been referred to as the library size in the literature.
2.Upper quartile (UQ) calculates the upper quartile of nonzero counts in each sample and uses its ratio over the average upper quartile across samples as the scaling factor.
3.Median calculates the median of nonzero counts in each sample and uses its ratio over the averaged median across samples as the scaling factor.
4.Trimmed mean of M values (TMM) takes the ratios of counts in a sample over the counts of an ad hoc reference sample and calculates a weighted trimmed mean of the log ratios across markers as the scaling factor. The *calcNormFactors()* function in the *edgeR* package was used for its estimation.
5.DEseq takes the ratios of the counts in a sample over the counts of a pseudo reference sample (defined as the geometric mean counts across samples) and calculates the median of the log ratios as the scaling factor. The *estimateSizeFactors()* and *sizeFactors()* functions in the *DESeq* package were used for its estimation.
6.PoissonSeq models the count data using a log-linear model and includes sequencing depth as a covariate in the model. The model is fitted using an iterative process that alternates between selecting equivalently expressed markers and estimating the model parameters. The *PS.Est.Depth()* function in the *PoissonSeq* package was used for its estimation.
7.Quantile normalization (QN) was inspired by quantile-quantile plot. This regression-based approach was originally developed for normalizing microarray data. It equates the rank statistics of each sample to the average rank statistics across samples. The *normalize.quantiles()* function in the *preprocessCore* package was applied to the count data after moderated logarithm transformation (ie, adding 1 to the count data to handling the zero counts before applying logarithm). With the relatively large proportion of zeros in the miRNA sequencing data, we have noticed a negative impact on the performance of QN due to the ambiguity in handling these tied zeros. As a result, we also examined the use of QN after filtering out markers with mean count < 4.
8.Surrogate variable analysis (SVA) is a regression-based approach that calculates the probabilities for markers being affected by the variation in sequencing depth and not by the biologic covariate of interest, uses the probability-weighted principal components to approximate the systematic variations in depth, and includes them as covariates in a linear model for the analysis of the log-transformed count data. The *svaseq()* function in the *sva* package was used. By default, it only includes markers that have > 5 reads in at least two samples.
9.Remove unwanted variation (RUV) is a regression-based approach that follows a similar overall strategy as SVA, aiming to use markers that have constant abundance levels across samples. It differs from SVA in several technical aspects, such as the use of the generalized linear model for the counts data in place of the linear model for log counts data. It is also available to use replicate samples for constructing covariates that represent systematic variations. The *RUVs()*, *RUVr()*, and *RUVg()* functions in the *RUVSeq* package were used, implementing the 3 variations of RUV.
